# A Bayesian Search for Transcriptional Motifs

**DOI:** 10.1371/journal.pone.0013897

**Published:** 2010-11-18

**Authors:** Andrew K. Miller, Cristin G. Print, Poul M. F. Nielsen, Edmund J. Crampin

**Affiliations:** 1 Auckland Bioengineering Institute, The University of Auckland, Auckland, New Zealand; 2 Department of Molecular Medicine and NZ Bioinformatics Institute, The University of Auckland, Auckland, New Zealand; 3 Department of Engineering Science, Faculty of Engineering, The University of Auckland, Auckland, New Zealand; Fondazione Telethon, Italy

## Abstract

Identifying transcription factor (TF) binding sites (TFBSs) is an important step towards understanding transcriptional regulation. A common approach is to use gaplessly aligned, experimentally supported TFBSs for a particular TF, and algorithmically search for more occurrences of the same TFBSs. The largest publicly available databases of TF binding specificities contain models which are represented as position weight matrices (PWM). There are other methods using more sophisticated representations, but these have more limited databases, or aren't publicly available. Therefore, this paper focuses on methods that search using one PWM per TF. An algorithm, MATCHTM, for identifying TFBSs corresponding to a particular PWM is available, but is not based on a rigorous statistical model of TF binding, making it difficult to interpret or adjust the parameters and output of the algorithm. Furthermore, there is no public description of the algorithm sufficient to exactly reproduce it. Another algorithm, MAST, computes a p-value for the presence of a TFBS using true probabilities of finding each base at each offset from that position. We developed a statistical model, BaSeTraM, for the binding of TFs to TFBSs, taking into account random variation in the base present at each position within a TFBS. Treating the counts in the matrices and the sequences of sites as random variables, we combine this TFBS composition model with a background model to obtain a Bayesian classifier. We implemented our classifier in a package (SBaSeTraM). We tested SBaSeTraM against a MATCHTM implementation by searching all probes used in an experimental *Saccharomyces cerevisiae* TF binding dataset, and comparing our predictions to the data. We found no statistically significant differences in sensitivity between the algorithms (at fixed selectivity), indicating that SBaSeTraM's performance is at least comparable to the leading currently available algorithm. Our software is freely available at: http://wiki.github.com/A1kmm/sbasetram/building-the-tools.

## Introduction

Identifying which transcription factors bind to which promoters is an important step towards understanding the transcriptional regulatory code. This identification process can be divided into two parts: determining the binding specificity of specific transcription factors, and then identifying TFBSs in a sequence using the binding specificity information.

There have been a number of papers proposing methods for one or both parts of the problem. Methods for finding transcription factors (as motifs which are statistically over-represented in sequences) can be broadly classified as those based on phylogenetic footprinting, and those which are not. These methods have been widely compared [1], [2] and reviewed [3]. The software implementations associated with many of these methods often also include software to use the motifs to identify TFBSs. For example, the popular motif finding software MEME [4] is packaged with the MAST software [5].

The link between determining binding specificity and finding sites where the transcription factor is likely to bind is the way in which binding specificity is represented. At present, the largest databases which are generally available, such as TRANSFAC [6] and JASPAR [7], represent binding specificity using an ungapped position weight matrix (PWM) representation. Each entry in an ungapped PWM, 
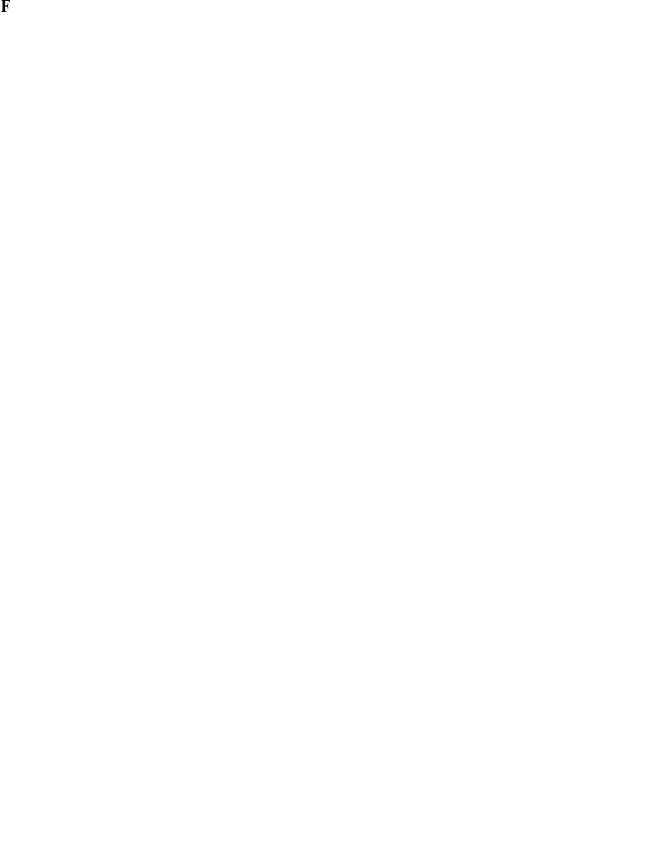
, is a weight for finding a particular base at a particular position from the start of the motif. There are several types of weights possible, but in this paper, we consider weights given as a raw count. At each aligned position 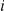 in the binding footprint, a frequency is recorded for each base 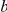 to give the matrix entry 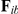. Let 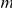 denote the total number of aligned sequences. Not all TFBS sequences are aligned to both ends, and so for each 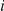, 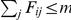. Note that in algorithms such as MEME, the algorithm alternates between finding an alignment, and determining the PWM, until the algorithm meets a termination condition and the final PWM is produced.

There are more sophisticated representations for transcription factor binding specificity, such as the Hidden Markov Model (HMM) approach used by MAPPER [8]. However, TRANSFAC and JASPAR collectively include a reasonably large number of matrices, and these are available to the public (albeit under commercial terms in the case of TRANSFAC). Other databases are either smaller in size, or as in the case of MAPPER, binding models are not available to the public (Voichita D. Marinescu, personal communication). For this reason, the focus of this paper is on methodology which uses only ungapped PWM information to search for transcription factor binding sites.

Representing transcription factor binding specificities in this form means that no data is stored on the interaction of binding specificity between different base positions in the binding sites. This is a reasonable approximation, as molecular binding models describing the interactions between transcription factors and DNA have shown that binding energies are approximately additive between bases [9] (in other words, interaction of binding specificity is negligible).

Existing PWM based search methodologies, such as MATCHTM, have not been justified based on a formal statistical model. MATCHTM instead computes scores using the formula [10]

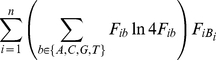
(1)where 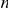 is the length of the matrix, 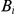 is the base at position 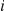 in the sequence, and 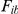 is the frequency of base 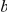 at position 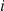.

## Methods

### Overall approach

Let 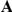 be a sequence of bases of length at least 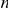 (where each base can be A, G, C, or T). We aim to make a decision about whether there is an exactly aligned TFBS starting at the beginning of 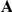.

We define a TFBS as a locus that is under evolutionary pressure so the sequence is one that a particular transcription factor will bind to. The sequence is used as evidence supporting the hypothesis that there is a TFBS at a particular locus. For example, the presence of a sequence exactly identical to the consensus sequence for the transcription factor is strong evidence for a TFBS. A sequence which is more distantly similar to the consensus sequence is weaker evidence for there being a TFBS. This is because there are an increasing number of possible sequences as the deviation from the consensus sequence increases, and so the null hypothesis that similarity to the consensus sequence arose by chance (as opposed to natural selection) becomes more credible.

Under this definition, a transcription factor either binds to a TFBS, or it does not; there is no attempt to model the degree of affinity, only to determine if there is evidence for an underlying process. Note that evolutionary pressure may select for a moderate TF-TFBS affinity, but against a stronger affinity. In this case, evidence for the TFBS is reduced, but may still be enough to detect the site.

We use two models of putative TFBS sequences. The foreground model describes the distribution of sequences under the alternative hypothesis that there is a TFBS at the site. The background model describes the distribution of sequences under the null hypothesis that there is no TFBS at the site.

### Foreground model

Our foreground model is best introduced in terms of a matrix of hidden parameters 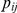 which represent the probability that a true TFBS will contain base 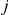 at position 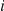. This parameter should not be confused with 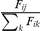, which is merely an estimator of 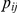. The true 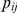 is unknown. For this reason, we build a statistical model of 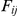, so we can express the joint distribution of 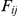 and the TFBS sequence, under the alternative hypothesis. We refer to the alternative hypothesis that this model applies as 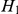.

Our foreground model requires that each base in a TFBS is independently selected in accordance with the hidden parameters. In practice, there are two ways in which new TFBSs are likely to arise. They may arise from convergent evolution, in which case the TFBS sequence is independent of all other TFBSs. Alternatively, an existing TFBS could be copied in a duplication event, creating a paralogous TFBS which is not independent of the original. Over time, however, mutations to less strongly conserved bases in the two TFBSs will reduce this dependence. For this reason, the independence assumption is reasonable except for very recently duplicated TFBSs.

If 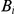 is the base at position 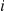 into a TFBS, the probability of the sequence 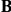 is
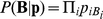
(2)


We assume, under this same model, that 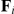 is a random variable produced by aligning 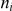 independent sequence samples (where 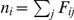), and therefore that
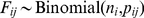
(3)


Hence,
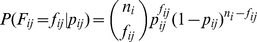
(4)where 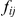 is a non-negative integer representing a frequency.

Now,
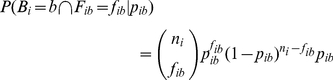
(5)

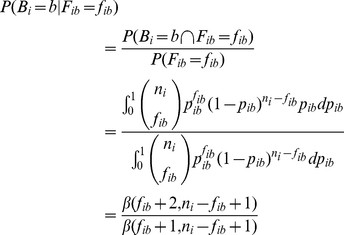
(6)where 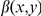 is the Euler Beta function [11].

Note that we assume that 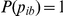 (*i.e.* without any samples from 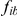, we know nothing about 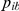). This is the same as the 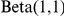 distribution, from the conjugate prior family to the Binomial distribution.

This gives us the ability to compute the probability of a given sequence under the alternative hypothesis:
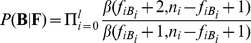
(7)


### Background model

We used a simple first-order Markov chain model, with one parameter for each base, 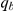, describing the probability that the base 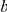 occurs at a particular point in the sequence. In addition, we introduce one parameter, 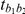 for each pair of bases 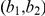, describing the conditional probability of finding base 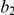 at a point in the sequence, given that 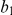 was present one base-pair earlier in the sequence. We refer to the null hypothesis that this model applies as 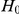.

We will assume that the foreground and background model are complementary. This is an approximation, because sequences might have higher order interactions not explained by either the foreground or background models. Making a simplifying assumption here is unavoidable because of the high complexity of these higher order interactions. For example, polypeptide coding sequences are considered background, and the distribution of the sequence of bases is determined by the effect of the polypeptide sequence on evolutionary fitness; something which would require more knowledge about biological function than is available, and is too complex to include in the background model.

However, the model nevertheless provides a principled approach for correcting for the length of the sequence, and for differences in the frequency of bases or pairs of bases. Hence,
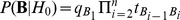
(8)


Recall that 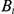 is the 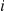
th nucleotide in the sequence being tested for a motif occurrence.

### Combining the models

In order to combine the foreground and background models, we start with Bayes' theorem:
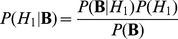
(9)


We assume the foreground and background models are complementary, so
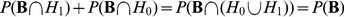
(10)

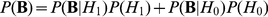
(11)


Due to complementarity,
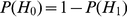
(12)


This leaves the prior probability 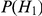 as the only remaining unknown. This should be an estimate of the rate of occurrence of the TFBS in the genome (or other set of sequences being searched). As this is not known, we make a plausible assumption about 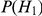, and later discuss the sensitivity of the accuracy of the method to this parameter.

We note that this combination of foreground and background models is able to represent a number of features to the extent that the information is present in the raw counts matrix. For example, gaps in the sequence correspond to regions in which the foreground is indistinguishable from the background, in which the value of 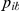 is identical to the probability of finding the base in the background. Similarly, palindromes can be represented merely by the incorporation of the palindromic pattern into 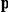. For this reason, there is no need for any special steps to be taken to allow BaSeTraM to find gapped or palindromic TFBS.

### Comparison with other work

Our model shares some similarities with the model used in a previous study [12]. However, we have taken a different approach at a number of points, as we discuss below. The most notable benefit of our approach compared to the Bayesian approach presented in the paper is that the approach of Lähdesmäki et al. requires a computationally expensive Markov Chain Monte Carlo (MCMC) procedure, while we can efficiently compute the posterior probability for a given motif being at a given position.

One major difference between the two approaches is that Lähdesmäki et al. aims to identify the posterior probability of alignments of one or more motifs in a given promoter region, while BaSeTraM computes the probability that a single motif is found at a given site, and uses this to annotate a sequence with probable sites. Another difference is that BaSeTraM does not take into account uncertainty in the background probabilities (and instead focuses entirely on the uncertainties in frequencies in the foreground model). This approximation can be justified by the large quantity of data available to build the background model (as opposed to the foreground models), and the correspondingly low estimator variance. Using this simpler background model allows BaSeTraM to efficiently use a context-dependent background model.

In addition, Lähdesmäki et al. used a different derivation, by representing all foreground model frequencies at each position using a four-way multinomial distribution across all bases. In this paper we instead use a binomial distribution, where one Bernoulli outcome is that a base at position 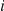 used to build the PWM row 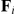 matches the base 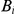, and the other is that it does not. In other words, we build a model of the motif matrix specific to 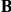, while Lähdesmäki et al. builds a general model. As discussed in the Implementation section, our formulation allows us to find a computationally efficient closed form solution (dependent on pre-computed values of the 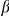 function) for the posterior probability.

### Implementation

We developed an implementation, SBaSeTraM, of the Bayesian search method, BaSeTraM, described above. We also implemented the method described in [10], and refer to the implementation as GMATIM. As the implementation of MATCHTM provided by the authors of that paper is closed source, GMATIM may differ from the BioBase MATCHTM implementation. For example, that paper stated that “the core of each matrix is defined as the first five most conserved consecutive positions of a matrix”. However, we have been unable to determine how the level of conservation of each group of 5 consecutive positions is measured and compared. To resolve this issue, we implemented GMATIM to simply find the 5 most conserved positions, where conservation at position 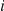 is measured as 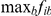.

In addition, we have created a wrapper, called WrapMAST, around the stand-alone MAST [5] binary, which we built from the MEME 4.4.0 source code (downloaded from http://meme.sdsc.edu/ on the 2nd of July, 2010). WrapMAST converts matrices from TRANSFAC into the form produced by MEME. This involves converting the matrix of frequencies to a matrix of log-odds 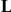. We have used the same formula used in the MEME software (using a background proportion of 0.25 for each base):
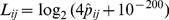
(13)

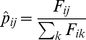
(14)


The addition of 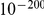 is used (as in MEME) in ensure that 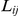 has a real value even when 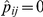. For each PWM, WrapMAST invokes MAST in hit list mode to search all probes. It then parses the output from MAST and outputs them in the same format used by SBaSeTraM (but with the p-value from MAST used in place of the posterior probability from SBaSeTraM).

SBaSeTraM, GMATIM, and WrapMAST are written in Haskell, and we have aimed to make the source code of each program a succinct and readable description of the corresponding algorithm. SBaSeTraM, WrapMAST, and GMATIM provide a similar command line interface (and share common code), so as to simplify the design of analyses which compare the algorithms.

Due to the possibility of numerical underflow from very small probabilities, our SBaSeTraM and GMATIM implementations make use of log probabilities (base 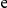).

It is necessary for SBaSeTraM to compute the posterior probability, 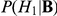, at every position in the sequence being searched, for every TFBS matrix (with the exception that there is no search for TFBS matrices of length 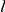 in a sequence of length 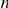 at starting positions 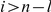). For this reason, it is important that the posterior probability can be computed efficiently.

The 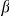 function has no closed form, and needs to be calculated numerically. To avoid expensive computations in our inner loop, for each matrix, we pre-compute 
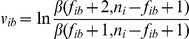
 for each 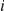 and 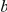. We also pre-compute all partial sums of the series 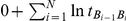, where 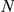 is the length of the sequence 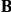. Let 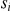 be the 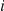
th entry in the series, so,
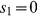
(15)

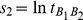
(16)

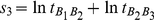
(17)and so on. This means that:
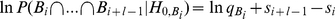
(18)

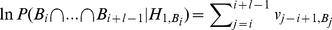
(19)


Note that equation 18 is a log-transformed equivalent of equation 8, and similarly, equation 19 is a log-transformed equivalent of equation 7.
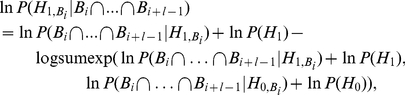
(20)where 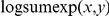 is a function which computes 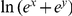 while avoiding numerical underflow for large magnitude negative values of 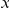 and 
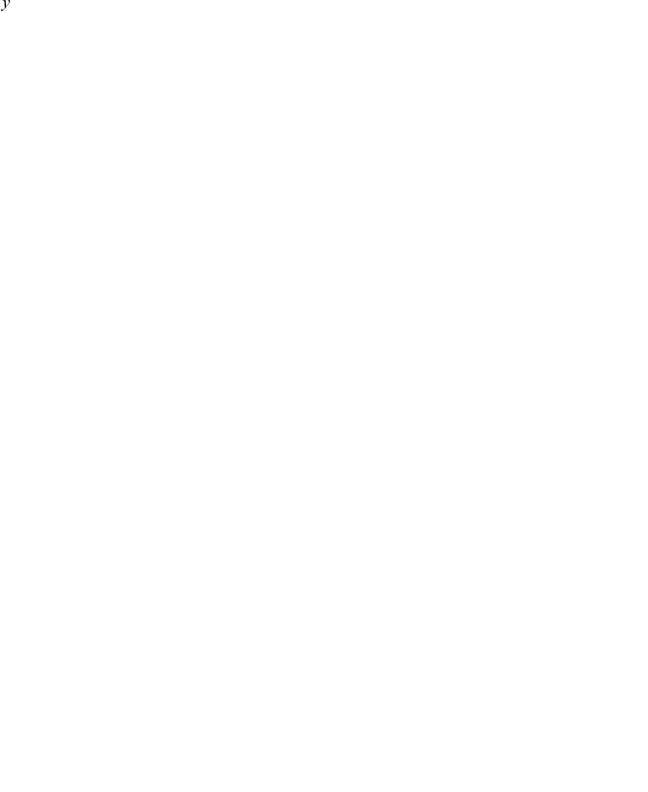
, by computing 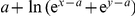 for 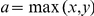.

We compute the vector 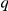 and the matrix 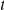 once, across all nucleotide sequences to be processed, by counting the number of occurrences of each base and sequence of the two bases, respectively, and dividing by the pooled total number of occurrences.

For each site, we compute the log-posterior probability and test it against a cut-off (as discussed below) to decide whether the TFBS occurs at that site. We search for sites, both on the sequences provided, and on the reverse complement of those sequences.

We retrieved the online supplement for [13] at http://fraenkel.mit.edu/Harbison/release_v24/. This data describes which of 6725 probes each of 182 different transcription factors bound to in a series of chromatin immunoprecipitation microarray (ChIP-chip) experiments. These probes were between 47 and 2764 base pairs long, with 95% between 92 and 1317 base pairs, 50% between 227 and 647 base pairs, and a median length of 359 base pairs. We also downloaded all TRANSFAC Saccharomyces Module matrices (TSM; [6]), as of 2009-06-16, from http://tsm.bioinf.med.uni-goettingen.de/.

Where a matrix used estimated rather than raw counts, as indicated by the occurrence of a decimal point in the ‘frequency’ matrix, that matrix was excluded (as we have assumed that raw counts will be used).

We filtered the set of probes, based on the experimental data, to only include those to which a transcription factor bound (for which we had a corresponding PWM). This left 1259 probes.

We then used each method to search the entire set of probes for TFBSs corresponding to each matrix, across all positions in the probe. Where the method detected the occurrence of a TFBS for a particular TF at any position in a probe, a positive result for that TF-probe combination was recorded. If no TFBSs were found at any position for a given TF a negative result was recorded. These results were then compared against the ‘gold standard’ experimental data. Only TFs which had corresponding matrices in TSM, and were also in the experimental results, were included.

We classified each included TF-probe pair into 4 categories:

- True Positive (TP) - positive prediction, and experimental determination of TF-probe interaction;
- False Positive (FP) - positive prediction, but no experimental determination of TF-probe interaction;
- True Negative (TN) - negative prediction, and no experimental determination of TF-probe interaction;
- False Negative (FN) - negative prediction, but experimental determination of TF-probe interaction.

In this paper we have used 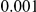 as an approximation of the prior probability, because this value is credible as a frequency of occurrences. To determine the sensitivity of this parameter, we tested values that were one order of magnitude higher, and one and two orders of magnitude lower. The posterior probabilities obtained from doing this are increased or decreased, respectively, but once this is taken into account when selecting cut-offs, there is very little difference in the results within this range of prior probability parameters.

## Results and Discussion

**Figure 1:**
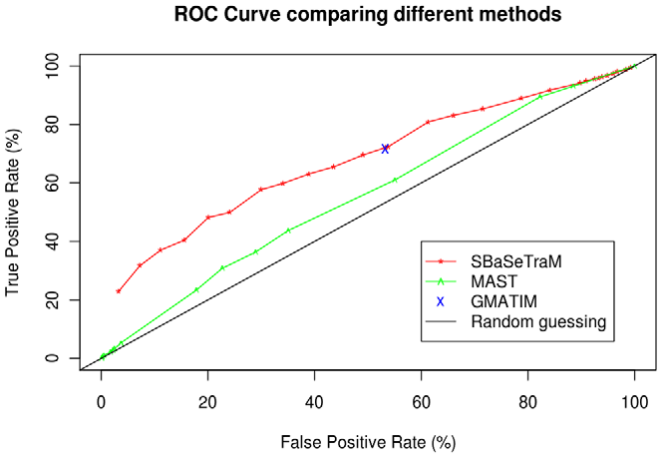
A receiver operating characteristics (ROC) curve comparing SBaSeTraM, GMATIM, and MAST. For SBaSeTraM, the posterior cut-off was varied to obtain a series of points. For MAST, the p-value cutoff was varied. For GMATIM, the parameters listed in the MATCHTM paper were used to generate the point on the curve.

There were 38 different transcription factors in TRANSFAC Saccharomyces Module, of which 32 were made up of raw counts. Of these, 16 were also found in the ChIP-chip dataset. These were tested against the 1259 different probes in the chromatin immunoprecipitation experiment. This gives 20144 different TF-probe pairs where we can classify whether the TF binds to the probe, and then check the classification. These results are shown in Figure 1.

We generated a ROC curve (Figure 1) for SBaSeTraM, by varying the posterior probability cut-off, and hence the trade-off between sensitivity and selectivity.

The point on the ROC curve generated using the parameters from [10] with GMATIM appears slightly below the ROC curve for BaSeTraM (GMATIM has 71.61% true positive rate for a 53.27% false positive rate). We found a posterior cutoff that generates a FPR close to this (with a posterior probability cut-off of 0.407, BaSeTraM achieved a 72.07% TPR at a FPR of 53.25%). At this point, we tested for a significant difference in the proportion of predictions which were correct; that is, 
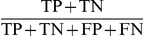
. We performed a comparison of these two binomial proportions, using the prop.test function in R [14], and obtained a one-sided p-value of 0.4603 (*i.e.* not significant to a 95% confidence level).

SBaSeTraM outperforms MAST when used through WrapMAST. It is worth noting that MAST is not typically used with TRANSFAC PWMs, and usually, multiple PWMs are used for each TF, and so the results cannot be used to make inferences about how well MAST performs together with MEME. The results do, however, illustrate the benefit of methods which take into account uncertainty in the foreground model.

**Figure 2:**
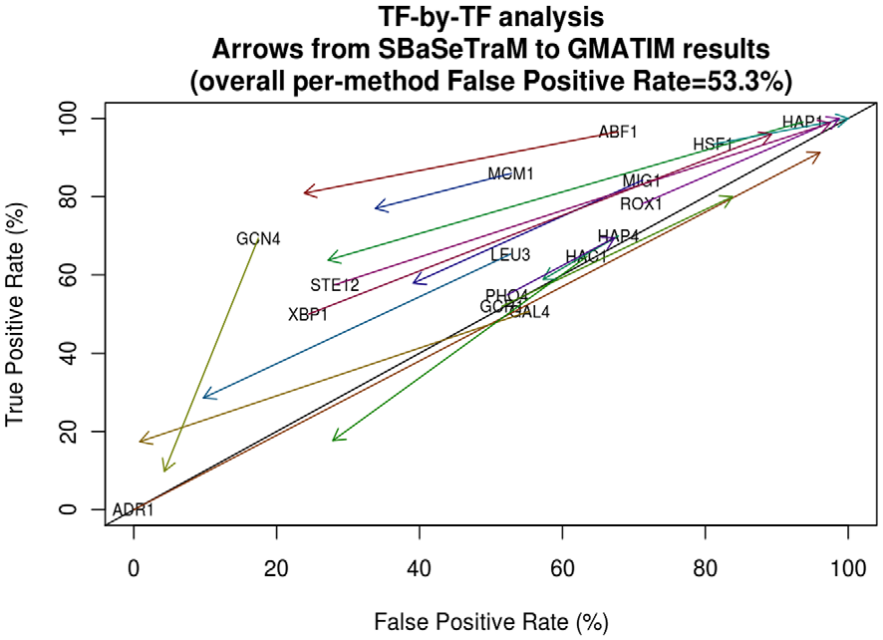
Comparing SBaSeTraM to GMATIM predictions for each transcription factor. The results are shown with the overall False Positive Rate for SBaSeTraM matched at that obtained from GMATIM with the parameters in the MATCHTM paper, namely 53.3%. Arrows run from the point obtained using SBaSeTraM to the point obtained using GMATIM.

We also carried out an analysis to see whether any particular TFs were making a large contribution to the overall prediction accuracy at this point. Figure 2 shows the differences between the two methods in the ROC space for each TF PWM. For each transcription factor, we have plotted an arrow from the point in the ROC space corresponding to the results for SBaSeTraM, to the point corresponding to the results from GMATIM. Some of the predictions are quite different; for example, for ADR1, SBaSeTraM found no occurrences, while GMATIM made numerous predictions, resulting in a true positive rate of 91.3% and a false positive rate of 96.0% (putting the accuracy for that particular TF below the line of no-discrimination). There was only one TF, GAL4, for which SBaSeTraM fell below the line of no-discrimination (which GMATIM predicted with a 17.4% true positive rate and a 0.8% false positive rate), and three TFs for which GMATIM fell below the line of no-discrimination (all of which were above or on the line of no-discrimination for SBaSeTraM). Unlike for SBaSeTraM, GMATIM predictions for HSF1, ROX1, and STE12 had true and false positive rates approaching 100%.

**Figure 3:**
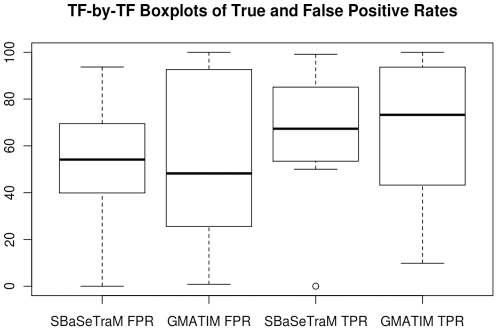
Box and whisker plot showing the spread of true and false positive rates for SBaSeTraM and GMATIM. The results are shown with the overall False Positive Rate for SBaSeTraM matched at that obtained from GMATIM with the parameters in the MATCHTM paper, namely 53.3%.

We also analysed the spread of true and false positive rates for each method. Figure 3 shows box-and-whisker plots for the true and false positive rates for SBaSeTraM and GMATIM. Notably, there is a much greater distance between the upper and lower quartiles in both the true and false positive rates for GMATIM than there is for SBaSeTraM. This demonstrates that the BaSeTraM algorithm is more consistently controlling the trade-off between sensitivity and selectivity for each individual TF.

In addition, we used the bisection method to find a separate posterior probability cutoff for each of the 16 TFs that gave the SBaSeTraM method a FPR (for that TF) close to the FPR obtained with GMATIM. We allowed the method to terminate when a cutoff was found that brought the 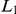 distance of the two FPRs within 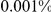, when an increase in cutoff resulted in an increased FPR (or a decrease in the cutoff resulted in a decrease in the FPR), or when no improvement in FPR was achieved after 4 iterations of the algorithm. The latter two conditions are necessary because there are a finite number of probes (1259), and there is no guarantee that there will be a cutoff which brings the SBaSeTraM FPR within 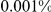 of the GMATIM FPR. In practice, for 8 of the 16 TFs, the difference between the final FPRs for the two methods was less than 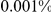, for 11 it was within 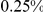, and for 13 was within 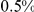. For HAC1, the final SBaSeTraM FPR was 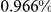 higher than the GMATIM one, for XBP1 the GMATIM FPR was 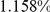 higher, and for HAP1, the final SBaSeTraM FPR was 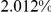 higher.

Using the same methodology used on the entire dataset (as discussed above), we tested for a statistically significant difference in proportion of predictions which were correct for each transcription factor, between GMATIM and SBaSeTraM (with the posterior probability cutoffs discussed in the previous paragraph). We obtained only one result where the p-value was less than 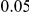, for GCN4 (p = 0.00886). For this TF, the FPR for both methods was 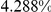, the TPR for SBaSeTraM was 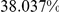, while it was 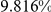 for GMATIM. When we applied the Holm-Bonferroni procedure for multiple comparisons [15], none of the TF-by-TF results were significant to a 5% familywise error rate (FWER).

### Conclusions

We have developed a Bayesian classifier for identifying TFBSs, which performs comparably to an existing algorithm, but which has a more principled statistical explanation, so that the trade-off between sensitivity and selectivity can be trivially adjusted, and the method can be altered to use different background models.

It is clear that the two methods are very similar in overall performance, and there is insufficient data in TSM to tell the two apart. The 95% confidence interval for the difference of the proportion correctly classified above runs from SBaSeTraM being 1.03% better, to GMATIM being 0.93% better. We therefore conclude that until there is more evidence that one method is better, from a performance standpoint, the two methods can be used interchangeably.

However, the fact that the statistical interpretation of BaSeTraM has been explained in rigorous terms, combined with the ease with which the posterior probability cut-off can be adjusted (as opposed to needing to adjust two separate parameters and re-run the analysis) makes the use of BaSeTraM preferable for many applications.

We note that despite the similarity in accuracy, the predictions made are not all the same; only 62.8% of all predictions of transcription factor binding made by SBaSeTraM with this posterior probability cut-off were also made by GMATIM.

The BaSeTraM statistical model includes a background model to be used. While a relatively uninformative background model is useful with the synthetic probes used in ChIP-chip analyses, using a different background model is likely to be important on genomic scale data, where there are localised variations in base frequencies.

When dealing with genomic scale data, it is also important that computation is reasonably efficient. It is also preferable that this computation can occur on modest hardware, so it is usable by groups without access to high-performance computing infrastructure.

In order to achieve these goals, we also developed a C++ implementation of BaSeTraM, called CBaSeTraM, which we optimised for the AMD64 architecture. We used Callgrind [16] to identify places where cache misses were occurring. We then used a customised allocator to ensure that all data which is needed in the inner loop (which is executed for each matrix for each alignment for each position) does not result in any cache misses, due to it being present in one cache page. As reading the level 1 and 2 caches are approximately 10 and 300 times faster than RAM, respectively, this leads to significant speed-ups. In this tool, we also implemented a sliding window determination of background model parameters 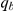 and 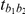. Our implementation supports two distinct sliding windows; the intention is that one window is much larger than the other. The final estimate of each 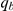 and 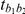 is the geometric mean of the two estimates. By default, the small window is 501 BP wide, and the large window is 2001 BP wide. Both windows are centred on the same base, which is used as the first position when testing for TFBSs. In addition, CBaSeTraM can use MPI [17] to search multiple sequences in parallel.

GMATIM, SBaSeTraM, and CBaSeTraM, as well as the programs used to test the methods, are Free/Open Source software. Instructions for building these programs are included as an online supplement.
